# Establishment and Biological Characterization of a Panel of Glioblastoma Multiforme (GBM) and GBM Variant Oncosphere Cell Lines

**DOI:** 10.1371/journal.pone.0150271

**Published:** 2016-03-30

**Authors:** Zev A. Binder, Kelli M. Wilson, Vafi Salmasi, Brent A. Orr, Charles G. Eberhart, I-Mei Siu, Michael Lim, Jon D. Weingart, Alfredo Quinones-Hinojosa, Chetan Bettegowda, Amin B. Kassam, Alessandro Olivi, Henry Brem, Gregory J. Riggins, Gary L. Gallia

**Affiliations:** 1 Department of Neurosurgery, Johns Hopkins University School of Medicine, Baltimore, MD, United States of America; 2 Johns Hopkins Physical Science Oncology Center and Institute for NanoBioTechnology, Johns Hopkins University, Baltimore, MD, United States of America; 3 Department of Pathology, Johns Hopkins University School of Medicine, Baltimore, MD, United States of America; 4 Department of Oncology, Johns Hopkins University School of Medicine, Baltimore, MD, United States of America; 5 Department of Neurosurgery, Aurora Neuroscience Innovation Institute, Milwaukee, WI, United States of America; University of Michigan School of Medicine, UNITED STATES

## Abstract

**Objective:**

Human tumor cell lines form the basis of the majority of present day laboratory cancer research. These models are vital to studying the molecular biology of tumors and preclinical testing of new therapies. When compared to traditional adherent cell lines, suspension cell lines recapitulate the genetic profiles and histologic features of glioblastoma multiforme (GBM) with higher fidelity. Using a modified neural stem cell culture technique, here we report the characterization of GBM cell lines including GBM variants.

**Methods:**

Tumor tissue samples were obtained intra-operatively and cultured in neural stem cell conditions containing growth factors. Tumor lines were characterized *in vitro* using differentiation assays followed by immunostaining for lineage-specific markers. *In vivo* tumor formation was assayed by orthotopic injection in nude mice. Genetic uniqueness was confirmed via short tandem repeat (STR) DNA profiling.

**Results:**

Thirteen oncosphere lines derived from GBM and GBM variants, including a GBM with PNET features and a GBM with oligodendroglioma component, were established. All unique lines showed distinct genetic profiles by STR profiling. The lines assayed demonstrated a range of *in vitro* growth rates. Multipotency was confirmed using *in vitro* differentiation. Tumor formation demonstrated histologic features consistent with high grade gliomas, including invasion, necrosis, abnormal vascularization, and high mitotic rate. Xenografts derived from the GBM variants maintained histopathological features of the primary tumors.

**Conclusions:**

We have generated and characterized GBM suspension lines derived from patients with GBMs and GBM variants. These oncosphere cell lines will expand the resources available for preclinical study.

## Introduction

Glioblastoma multiforme (GBM) is the most common primary malignant adult brain tumor [1]. The standard treatment regimen includes surgery, radiation, and chemotherapy [2]. Despite advances in this therapeutic regimen, treatment usually fails due to a combination of chemo- and radio-resistance and the intrinsic ability of the malignant cells to disperse widely through normal brain tissue, making complete surgical resection nearly impossible. Given the overall poor prognosis, with a median survival of only about 15 months [2], there is a significant need to develop improved therapeutic options for these patients.

Tumor cell lines are a vital part of cancer research as they serve as the primary model system for *in vitro* and *in vivo* experimentation. These models are utilized to study the basic cellular and molecular biology of tumors and are the basis for preclinical testing of new treatment modalities. Cell lines used for GBM research can be broadly subdivided into two groups depending on their *in vitro* growth conditions. First are adherent cell lines, which have been used widely in neuro-oncology research. These cell lines grow adherently on culture plates and are generally grown in serum [3–7]. Adherent cell lines have several important limitations as a drug discovery tool. They grow as compact masses *in vivo* and often do not infiltrate normal brain parenchyma [8, 9]. Furthermore, gene expression in adherent lines frequently does not correlate with gene expression in the source tissue [9].

In contrast to adherent cell lines, oncosphere lines (also called neurosphere or stem-like cell lines) are grown in culture conditions originally developed for neural stem cells—as free-floating spheres in serum-free medium supplemented with selected growth factors [8, 10–13]. Oncosphere-based models more closely resemble the original patient tumors, both histologically and genetically [9, 14]. For instance, Caldera *et al*. demonstrated that oncosphere cultures *in vitro* demonstrated similar genetic alterations, including EGFR amplifications, MGMT hypermethylation status, TP53 mutational status, and PTEN mutational status, as the original patients’ tumors [14]. Adherent lines established from the same patients did not retain all of these alterations. An earlier study by Lee *et al*. showed similar results when comparing the genotypic and phenotypic characteristics of suspension and adherent cell lines derived from the same tissue source [9]. Moreover, several groups have shown that GBM oncospheres grown in an orthotopic xenograft model retain the histopathological features of GBMs [8–10, 14–17]. Given these characteristics, oncosphere lines are more suitable models for preclinical purposes [8–10, 14–18].

To date, the adoption of oncosphere lines as model systems has been hampered by the paucity of established lines. Interestingly, no GBM oncosphere lines are currently included in the American Type Culture Collection. In this paper, we established and characterized 13 primary GBM oncosphere cell lines from fresh surgical specimens. These lines include rare variants, such as GBM with embryonal (PNET) and oligodendroglial features. We believe that such cell lines derived from our and other culturing protocols could serve as more accurate *in vitro* and *in vivo* models of GBMs and therefore be an important tool in investigating treatment options for patients with malignant gliomas.

## Material and Methods

### Clinical information

Patients with suspected GBMs or previously diagnosed GBMs were identified prior to surgery at Johns Hopkins Hospital (JHH). Patient demographics, clinical follow-up, and pathology reports, including molecular analyses when performed, were obtained from the Electronic Patient Record system and Pathological Database System under Johns Hopkins Hospital Institutional Review Board approved protocols, NA_00001600 and NA_00035748.

### Cell line establishment

Tumor tissue was collected from the operating room during resection with written patient consent, under Johns Hopkins Hospital Institutional Review Board approved protocols, NA_00001600 and NA_00035748. Tissue was obtained from regions of viable tumor. Within 2 hours, the sample was transported to the laboratory on ice in 0.9% (w/v) NaCl. Under sterile conditions, approximately 50 mg of tissue was rinsed in PBS (Gibco, Grand Island, NY). Regions of necrosis, obvious vessels, clotted blood, and charred tissue were removed. The tissue was then grossly dissociated using two scalpels until it was close to liquid consistency.

This liquefied sample was then moved to a glass Dounce homogenizer and 500 μL of Minimal Essential Media (MEM) (Gibco) was added. The tissue was gently dounced until no obvious tissue fragments were visible. The resulting slurry was passed at least three times through a 16-gauge needle and then subjected to enzymatic dissociation by incubation with 50 μL of collagenase IV (10 mg/mL in HBSS) (Invitrogen #17104–019, Carlsbad, CA) at 37°C for 15 minutes.

The sample was then passed through a pre-wetted 70-micron filter to remove cell clumps and the filter was washed with 500 μL of MEM. Next, 3 mL of room-temperature red blood cell lysis buffer (BD #555899, San Diego, CA) was added and the specimen vortexed for 5 seconds. This was covered and incubated at room temperature for 15 minutes.

The cells were then centrifuged at 180 x g for 5 minutes at 4°C and resuspended in 1 mL of sterile-filtered PBS with 1% (v/v) fetal bovine serum (Gemini, Sacramento, CA). One mL of 30% (w/v) sucrose in deionized H_2_O was added to the cell suspension and the sample was centrifuged at 3,100 x g for 20 minutes at 4°C. The supernatant was discarded and the cell pellet was resuspended in 1 mL of NeuroCult medium (StemCell Technology #05751, Vancouver, BC, Canada) supplemented with 0.0002% heparin (StemCell Technology #07980), hEGF (final concentration 20 ng/mL, Peprotech #AF-100-15, Rocky Hill, NJ), and hFGF-b (final concentration 10 ng/mL, Peprotech #100-18B). The cell pellet was triturated to break up any remaining cell clumps. JHH-505 and JHH-520 were not subjected to red blood cell lysis and were resuspended in NeuroCult media following enzymatic dissociation and filtering.

Viable cells were counted using Trypan Blue exclusion (Sigma, St. Louis, MO) and a hemocytometer. Between 50,000 and 100,000 cells were removed from the solution and placed in a T25 cell culture flask with 5 mL of supplemented NeuroCult medium. The culture was placed in a humidified incubator at 37°C with 5% CO_2_.

Cultures were fed 1 mL of supplemented NeuroCult medium every 48 hours and monitored daily for development of oncospheres. Once oncospheres were observed, the flask contents were spun down at 180 x g for 5 minutes at 4°C and triturated into a single cell suspension. The cells were counted and resuspended in 10 mL of supplemented NeuroCult medium in a T75 tissue culture flask at a concentration of 5,000–10,000 cells/mL. At the fourth passage, the cell line was considered stable enough for further validating experiments.

### Long term cryogenic storage and recovery

For long term cryogenic storage, one T75 flask of cells was centrifuged at 180 x g for 5 minutes at 4°C. The resulting pellet was triturated in 1 mL of NeuroCult medium to obtain a single cell suspension. The cell slurry was then mixed with 1 mL of freezing solution, consisting of NeuroCult medium plus 10% (v/v) DMSO (Sigma). The solution was then transferred to a 2 mL cryogen tube (Thermo, Pittsburgh, PA) and frozen at -80°C. After 24 hours the tubes were moved to liquid nitrogen for long term storage. For cell recovery, cryovials were placed in a water bath at 37°C until thawed. The cell solution was then rapidly added to a T75 flask containing 10 mL of NeuroCult medium. The flask was then transferred to a humidified incubator at 37°C with 5% CO_2_.

### Short tandem repeat profiling

Genomic DNA was extracted from the cell lines using QIAGEN DNeasy Kit (Qiagen, Valencia, CA) following the manufacturer’s instructions. The DNA concentration was quantified using a Nanodrop 2000 (Thermo) and a minimum of 500 ng of genomic DNA was aliquoted at a concentration of 10 ng/μL. The short tandem repeat (STR) profile was performed at the Johns Hopkins University Core Fragment Analysis Facility using a StemElite ID System (Promega, Madison, WI).

### Cell line doubling times

Cells were triturated into a single-cell suspension and viable cells counted using Trypan Blue exclusion and a hemocytometer. One hundred thousand cells were resuspended in 10 mL of supplemented NeuroCult medium and fed 2 mL of medium every 3 days. On day 15, the cultures were spun down, the spheres were triturated, and the total viable cell number was counted again using Trypan Blue exclusion and a hemocytometer. Doubling times were then calculated using an online calculator [19].

### Multipotency

Oncospheres were triturated into a single-cell suspension and then counted using Trypan Blue and a hemocytometer. Between 5,000 and 15,000 cells/cm^2^ were plated on poly-D-lysine/laminin coated glass coverslips (BD #354087) in a 24 well plate with 500 μL of NeuroCult medium supplemented with 0.0002% heparin and 10 ng/mL of hFGF-b. After 3–5 days, when the cells were visibly adhered to the coverslips, the media was aspirated and replaced with 500 μL of NeuroCult medium supplemented with 1% fetal bovine serum. Five hundred microliters of medium was added every 3–4 days for a minimum of 10 days, until morphological changes were noted. The cells were then stained with 1:500 rabbit anti-GFAP (Dako #Z0334, Carpinteria, CA) or 1:500 mouse anti-Tuj1 (Covance #MMS-435P, Princeton, NJ) and secondary antibody [1:500 goat anti-rabbit and goat anti-mouse (Invitrogen #A-11008 and #A-21124)].

### *In-vivo* tumor formation

*In vivo* tumor formation was assayed using 5- to 6-week-old female athymic nude mice (NCI, Frederick, MD) as described previously [20]. All animal experiments were carried out under a Johns Hopkins University Animal Care and Use Program approved protocol (MO09M429). Briefly, 1.5x10^5^-1x10^6^ cells were injected stereotactically into the striatum of two to four animals. Animals were monitored weekly until development of neurological deficits, at which point they were euthanized or until they passed away from natural causes. Brains were removed, fixed in formalin for at least 24 hours, and stained with hematoxylin and eosin (H&E). Each mouse brain was examined and characterized by board certified neuropathologists (BAO and CGE). In cases from patients with GBM variants, xenografts were also compared to H&E slides from the original patient tumors.

## Results

### Clinical histories

All 12 patients from whom cell lines were established carried a diagnosis of either a GBM or GBM variant. Clinical data from these patients are summarized in Table 1. All patients were adults ranging from 22 to 76 years old.

**Table 1:** Clinical data of the patients.

Cell Line	Gender	Diagnosis	Location	Prior Therapy	Survival* (Months)
*JHU-0879*	F	GBM, with PNET-like component	Left Parieto-occipital Lobe	None	10
*JHH-66*	F	GBM	Right Occipital Lobe	None	3
*JHH-75*	F	GBM	Left Frontal Lobe	None	43
*JHH-136*	M	GBM	Right Frontal Lobe	None	17
*JHU-0937*	M	GBM	Left Temporal Lobe	None	12
*JHH-211*	M	GBM	Left Frontal Lobe	None	9
*JHH-227*	M	GBM	Right Periatrial Region	None	Lost to follow-up 6 months after surgery
*JHH-245*	M	GBM	Right Occipital Lobe	None	14
*JHU-1014*	M	GBM	Left Temporal Lobe	XRT, TMZ + Bevacizumab	5
*JHU-1016A*	M	GBM	Right Temporal Lobe	None	18
*JHU-1016B*	M	GBM	Right Temporal Lobe	None	18
*JHH-505*	M	GBM with oligo component	Right Parietal Lobe	None	40
*JHH-520*	F	GBM	Right Frontal Lobe	None	2

*: Survival calculated from date of tissue acquisition.

XRT: Radiotherapy.

TMZ: Temozolomide.

Cell line JHU-0879 was derived from a female patient with a tumor in the left parieto-occipital lobe. The lesion was found to be a GBM with areas of PNET features [21]. After surgical resection the patient received adjuvant radiotherapy and temozolomide, an alkylating agent, followed by three cycles of temozolomide. The patient recurred 7 months after surgery and she passed away 10 months after her initial surgery.

Cell line JHH-66 was derived from a female patient who presented with a GBM in her right occipital lobe. She demonstrated radiographic progression within 1 month of surgery and passed away 3 months after her tumor resection.

Cell line JHH-75 was derived from a female patient with a GBM in her left frontal lobe. After resection, the patient received concurrent radiotherapy and temozolomide followed by 6 cycles of adjuvant temozolomide. After progression, the patient received a second resection followed by 1 cycle of BCNU, an alkylating agent. She progressed through BCNU and underwent a third resection. At that point, the patient enrolled in a clinical trial with a PDGF monoclonal antibody, IMC-3G3. After 4 cycles she progressed and underwent a fourth resection with placement of Gliadel wafers, surgically-implanted, BCNU-impregnated, biodegradable wafers. Tumor regrowth led to a fifth resection, at which time MGMT was reported to be methylated, which was followed by concurrent radiotherapy and temozolomide. She subsequently had a sixth resection for progression. The patient received no additional therapies and died 43 months after her diagnosis of GBM.

Cell line JHH-136 was derived from a male patient with a GBM in the right frontal lobe. He was lost to follow-up but, according to the Social Security Death Index, died 17 months after his initial surgery.

Cell line JHU-0937 was derived from a male patient with a GBM in the left temporal lobe. Following surgery, the patient received adjuvant radiotherapy and temozolomide in addition to going on a clinical trial for veliparib, a PARP inhibitor. He continued to take veliparib during his adjuvant temozolomide for four cycles. After his fourth cycle, the patient progressed and enrolled in a clinical trial with cediranib, a VEGF inhibitor, and cilengitide, an integrin α_V_ inhibitor. The patient progressed after his third cycle and was removed from trial. He then received one dose of bevacizumab, an anti-VEGF antibody, and passed away 12 months after surgery.

Cell line JHH-211 was derived from a male patient with a tumor in the left frontal lobe. The tumor was diagnosed as an anaplastic astrocytoma by biopsy. Upon resection one month later, the tumor was diagnosed as GBM. Tissue for culture was obtained from the resection. After surgery, the patient received concurrent radiotherapy and temozolomide followed by one cycle of adjuvant temozolomide. The patient died 9 months following resection.

Cell line JHH-227 was derived from a male patient with a right periatrial GBM. He received concurrent radiotherapy and temozolomide following surgery. The patient was lost to follow-up 6 months after surgery.

Cell line JHH-245 was derived from a male patient with a GBM in his right occipital lobe. He received Gliadel at surgery and afterwards received concurrent radiotherapy and temozolomide. Following this, he received 2 cycles of adjuvant temozolomide before progressing. The patient then underwent an additional resection at which time tissue was removed for a clinical trial involving Vitespen, a heat shock protein-peptide complex-96 based vaccine. After his second surgery the patient was put on bevacizumab in addition to the vaccine treatments. He received this treatment for 4 months before progressing and died 14 months after his initial surgery.

Cell line JHU-1014 was derived from a male patient with a recurrent GBM in the left temporal lobe. The initial diagnosis of GBM was made following surgical resection 12 months prior to obtaining tissue. In the interval, the patient received 6 weeks of radiotherapy with concomitant temozolomide and bevacizumab. Following seven cycles of temozolomide and bevacizumab, progression prompted additional surgical intervention. Tissue obtained from this procedure, which was notable for extensive treatment effect on histopathological analysis, was used for cell line establishment. The patient died 5 months later.

Cell lines JHU-1016A and JHU-1016B were derived from a male patient with a GBM in his right temporal lobe. The specimen obtained from surgery was split into two pieces, which were used to create two separate cultures. After resection the patient received concurrent radiotherapy and temozolomide. The patient then received adjuvant temozolomide with bevacizumab for 9 cycles before progressing. He then switched to CCNU, an alkylating agent, and bevacizumab for one cycle. He died 18 months after his initial surgery.

Cell line JHH-505 was derived from a male patient with a GBM with oligodendroglioma component in his right parietal lobe. Molecular studies reported partial deletions on chromosomes 1p and 19q. The 1p deletion did not include FUBP1 but the region of loss on 19q did include CIC, both oligodendroglioma-implicated genes [22]. The patient returned to his home country for additional treatment and passed away 40 months after tissue was obtained.

Cell line JHH-520 was derived from a female patient with a right frontal GBM. She received her initial diagnosis from a recent resection at an outside hospital. Tissue was obtained from her second surgery, 9 days later, at which time the diagnosis of GBM was confirmed; MGMT was not methylated. The patient passed away 2 months after surgery.

### Tissue samples

**Fig 1:**
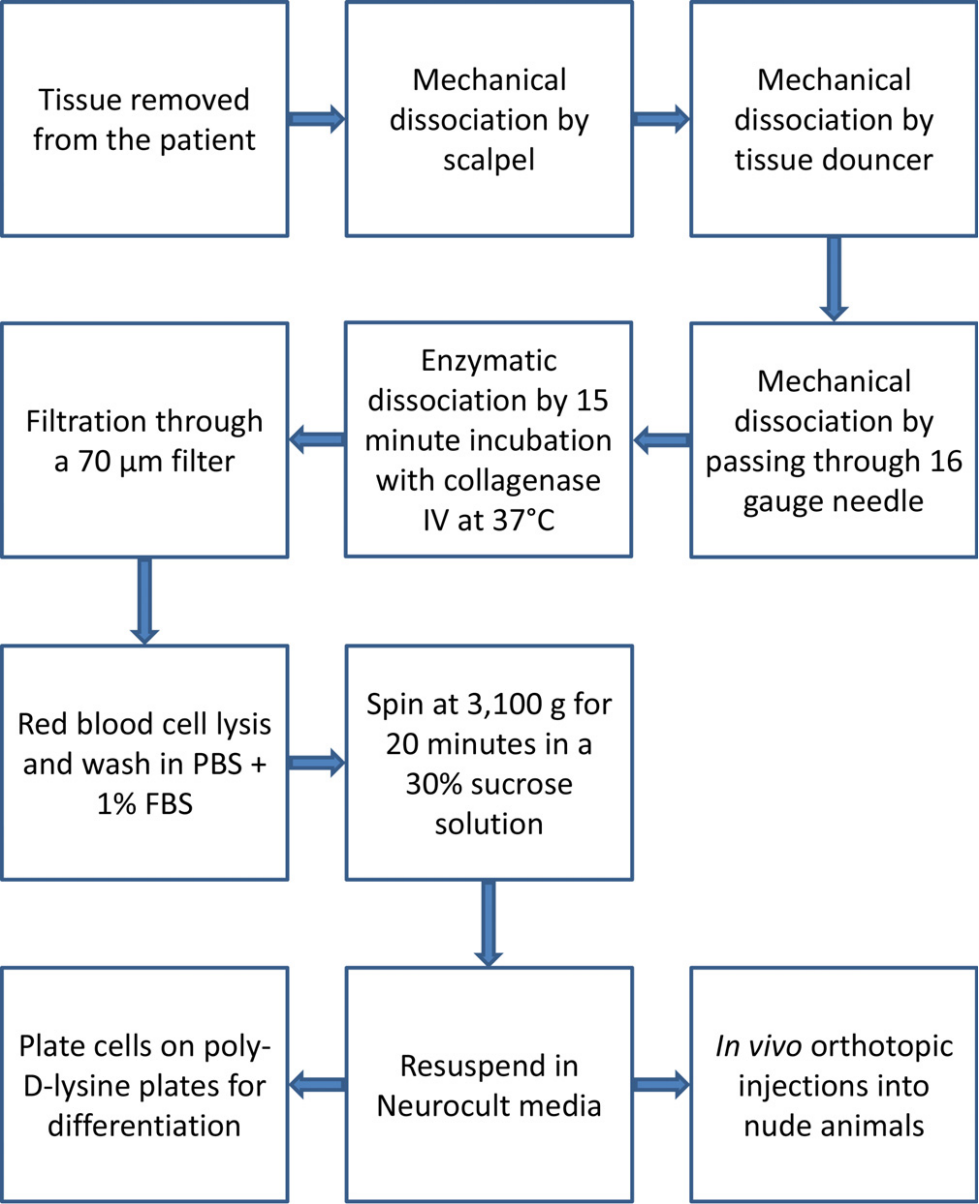
Summary of tissue processing and cell line establishment protocol.

Over an 18-month period, approximately 50 tumors were processed. Out of these samples, we established 13 suspension cell lines from 12 patients. Two of these lines were subclones from a single patient (JHU-1016A and JHU-1016B). Multiple additional tissue samples resulted in short-term suspension cultures; however, these cultures did not grow past passage four and were not analyzed. Each cell line was prepared using a fixed protocol (Fig 1). We evaluated the cell lines using four criteria including: 1) genetic uniqueness using STR profiling; 2) oncosphere formation/self-renewal past passage four; 3) multipotency as demonstrated by differentiation; and 4) *in vivo* orthotopic tumor formation (Table 2).

**Table 2:** Cell line establishment criteria.

Cell line	STR profiling	Doubling time	Multipotency	*In vivo* tumor formation	# of cells injected/ Average survival (Days)
*JHU-0879*	+	Fast*	+	+ 1/1^a^	5x10^5^/87^b^
*JHH-66*	+	Slow	+	+ 3/3	2.5x10^5^/144
*JHH-75*	+	Slow	ND	ND	ND
*JHH-136*	+	Fast*	+	+ 3/3	6x10^5^/161
*JHU-0937*	+	Fast	+	- 0/4	1x10^5^/NA
*JHH-211*	+	Fast	+	+ 3/3	2x10^5^/389
*JHH-227*	+	Slow*	+	+ 3/3	2x10^5^/71
*JHH-245*	+	Fast	+	+ 4/4	1.5x10^5^/89
*JHU-1014*	+	Slow	ND	- 0/1^a^	5.55x10^5^/NA
*JHU-1016A*	+	Fast	+	+ 4/4	2.5x10^5^/108
*JHU-1016B*	+	Slow*	+	+ 4/4	2.5x10^5^/194
*JHH-505*	+	Fast	ND	+ 3/3	5x10^5^/193
*JHH-520*	+	Fast	ND	+ 2/2	5x10^5^/84

STR: Short Tandem Repeat.

ND: Not done.

NA: Not applicable.

+: Positive for tumor.

-: Negative for tumor.

*: Quantified doubling time.

^a^: Two other animals died but were unable to be evaluated for tumor formation.

^b^: Survival reported is from an n of 1.

### Cell line independence

**Fig 2:**
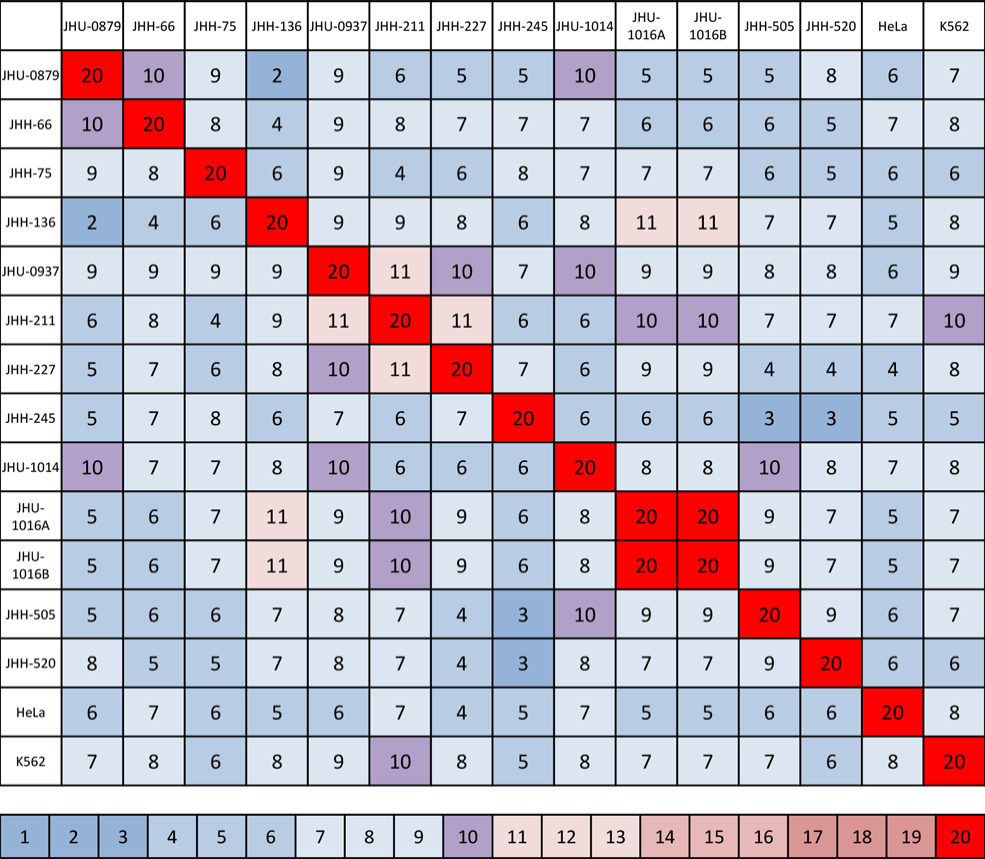
Outcome of short tandem repeat DNA profiling of the established cell lines showed the unique profile of each line. Twenty markers were assayed and an overlap of 16 or more markers (80%) was considered an indication of cross-contamination. HeLa and K562 were included both as quality controls and to show lack of contamination from these ubiquitous cell lines.

To ensure cell line independence, we performed STR profiling (Fig 2). An overlap of 80% was considered non-coincidental and suggestive of a shared primary source [23]. With the exception of JHU-1016A and JHU-1016B, none of the lines showed significant genetic overlap. JHU-1016A and JHU-1016B were established from the same tumor specimen and were, as anticipated, identical with respect to their STR profiles. Among the remaining lines, the overlap was minimal, ranging from 0 to 40%. STR profile markers are shown in S1 Table. The cell lines’ STR profiles were also compared to 15 established astrocytoma cell lines from ATCC. Overlap ranged from 17 to 61%, below the 80% threshold and confirming the genetically unique nature of each new cell line established.

### Doubling times

**Fig 3:**
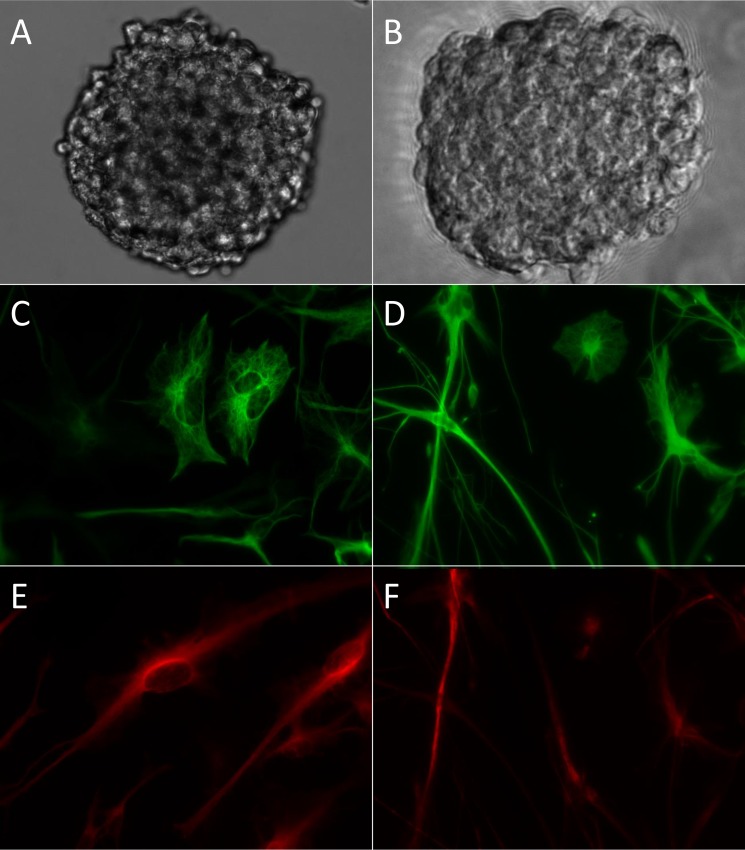
Cell line growth and multipotency. (A-B) Phase contrast images of oncospheres from *JHU-0879* (A) and *JHU-0937* (B). (C-F) Immunofluorescence images demonstrating the multipotency of the oncospheres. (C-D) Astrocytic-like cells stained for glial fibrillary acidic protein in lines *JHU-0879* (C) and *JHU-0937* (D). (E-F) Neuronal-like cells stained for class III β-tubulin in lines *JHU-0879* (E) and *JHU-0937* (F). All images taken at 400x.

All 13 cell lines demonstrated growth of spheres beyond passage four (Fig 3A and 3B). We observed the cell lines grew at different rates. They all grew noticeably slower than U87, one of the most widely used adherent GBM cell lines [24]. To quantitate this variability, we performed proliferation assays on several lines. The cell lines were separated into two groups based on their doubling times. The slower cell lines had doubling times of 4.5 days and greater. The faster group had doubling times 2.5 days and shorter. Based on the doubling times of these 4 lines, we subjectively grouped the remaining 9 cell lines as either slow- or fast-growing (Table 2).

### Multipotency and differentiation

Similar to neural stem cells, GBM cells grown as oncospheres retain the ability to differentiate down multiple cellular lineages [8]. To demonstrate the multipotency, we performed *in vitro* differentiation assays on 9 lines (Table 2). Two of the remaining cell lines, JHH-75, and JHU-1014, were not assayed due to their slow growth characteristics. The final cell lines, JHH-505 and JHH-520, are newer lines and have not yet been analyzed. After morphological changes were observed, the cells were stained for glial fibrillary acidic protein (GFAP) and class III β-tubulin (Tuj1) to evaluate multipotency (Fig 3). Differentiation showing markers of two lineages from a single population demonstrates multipotency of the oncospheres. Upon differentiation, we observed the presence of the glial marker, GFAP (Fig 3C and 3D), and the neuronal marker, Tuj1 (Fig 3E and 3F), in all 9 oncosphere lines evaluated.

### *In vivo* tumor formation

We next evaluated *in vivo* tumor formation. Using orthotopic injections of cells into immune-deficient mice, we demonstrated the capabilities of these cells to form tumors. The time to euthanasia varied between 71 and 389 days (Table 2). Of the 12 cell lines injected into animals, 9 formed tumors in all injected animals. In JHU-0879, one of three animals developed a tumor. The other animals in the group passed away but were unable to be evaluated for tumors on necropsy. In JHU-0937, the animals died of other causes and on necropsy there was no evidence of tumor. In JHU-1014, one animal passed away of other causes with no tumor evident on necropsy and the other two animals were unable to be evaluated for tumors on necropsy. JHH-75, has not yet been subjected to *in vivo* tumor formation assessment due to its slow growth rate.

**Fig 4:**
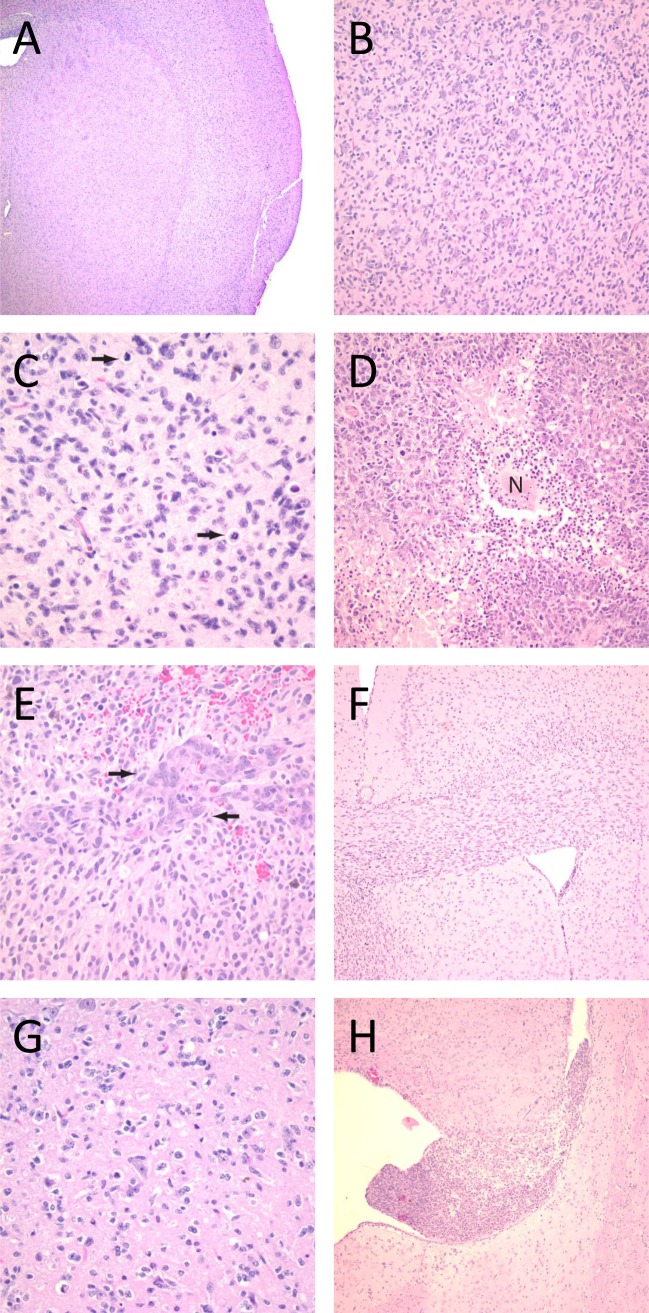
H&E stains of orthotopic tumors formed in athymic mice, showing hallmark characteristics of GBMs. (A and B) All of the lines showed diffuse invasion of normal parenchymal. The hallmark features of glioblastoma were identified in the brains of mice injected with oncosphere lines, including increased mitotic activity (C, arrows), necrosis (D, designated “N”), and vascular proliferation (E, arrows). The oncosphere lines also demonstrated other histologic features specific to invasive gliomas, including spread through white matter tracts (F), neuronal satellitosis (G), and subventricular tumor formation (H). Original magnification for panel A was 25x; for F and H, 50x; for B and D, 100x; and for C, E, and G, 200x.

Pathological evaluation showed some heterogeneity between the lines. All the xenografts contained cells with angulated or elongated nuclei and hyperchromasia, characteristic of astrocytomas. All of the xenografts generated showed diffuse invasion into brain parenchyma (Fig 4A and 4B). Furthermore, the standard histologic criteria used to diagnose GBM were identified among the different lines, including increased mitotic activity (Fig 4C), necrosis (Fig 4D), and vascular proliferation (Fig 4E). While significant mitotic activity was seen in all cases, the presence of vascular proliferation and necrosis was variable. The invasive growth was very prominent in many of the lines, including invasion of the corpus callosum and other white matter tracts (Fig 4F). This invasion is characteristic of infiltrating gliomas but often absent in adherent models [8, 9]. Some xenografts demonstrated other features specific to invasive glial neoplasms including neuronal satellitosis (Fig 4G) and subventricular tumor formation (Fig 4H). Taken together, the classic histopathological features of GBM were well-represented in this cohort of tumor xenografts.

**Fig 5:**
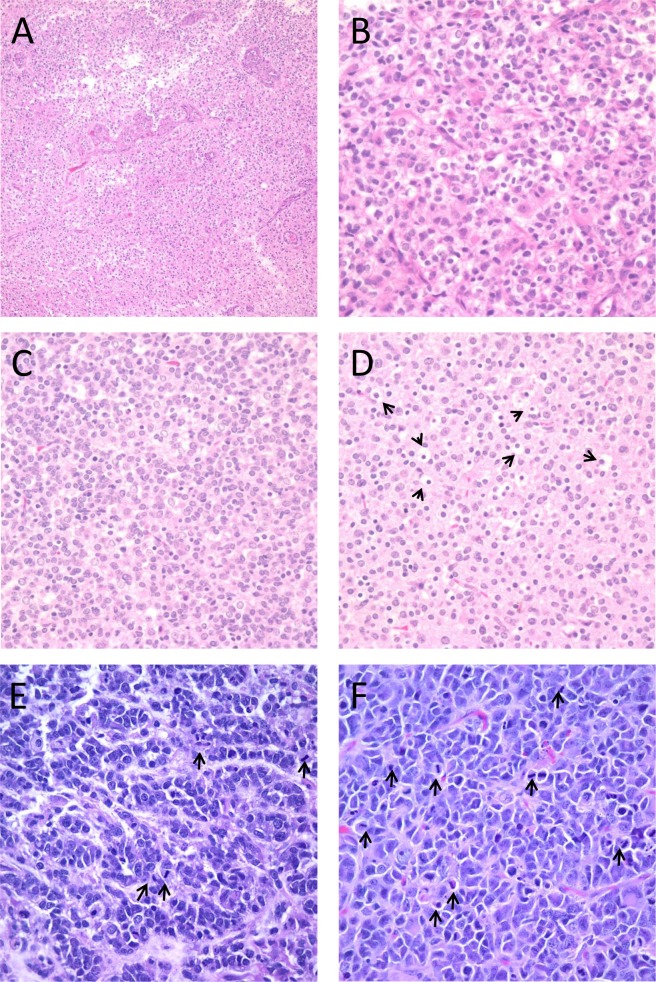
H&E stains of primary tumor tissue and orthotopic tumor from the GBM variant cell lines showing specific features of each variant. The JHH-505 primary tumor has features of a GBM such as vascular proliferation and dense cellular areas (A) while certain areas contain round, regular oligodendroglial appearing cells (B). The JHH-505 xenograft tumor also contains histological characteristics consistent with GBM-O, including dense (C) and less cellular (D) regions with oligodendroglial appearing cells. The JHU-0879 primary tumor has areas consistent with PNET with hypercellularity containing cells with scant cytoplasm and prominent nucleoli (E). The JHU-0879 xenograft also has PNET features, such as densely packed cells and prominent nucleoli (F). Arrows highlight mitotic or apoptotic cells. Magnification for panel A is 100X, panel B-F are 400X.

In cases of cell lines from patients with GBM variants, the xenografts were also compared to the primary patient tumor. JHH-505 was isolated from a GBM with an oligodendroglioma component (GBM-O). In addition to hypercellularity and neovascularization (Fig 5A), this primary tumor contained many small, round, regular cells with surrounding clear halos indicative of oligodendroglial differentiation (Fig 5B). The JHH-505 xenograft retained features of a mixed GBM, with a dominant astrocytic component (Fig 5C) but also numerous scattered oligodendroglial-like cells which were prominent in some areas (Fig 5D). JHU-0879 was derived from a GBM with PNET features. The primary tumor contained areas of densely packed cells with scant cytoplasm and prominent nucleoli (Fig 5E). The xenograft contained these features as well but the nucleoli were more prominent in the xenograft than in the primary tumor (Fig 5F). Throughout both tumors were an abundance of mitotic and apoptotic cells. This data further validates the notion that oncosphere cell lines often recapitulate the histopathological features of the original patient tumor.

## Discussion

Despite recent advances in treatment modalities, GBM remains a cancer with a very poor prognosis [25]. Cell lines that retain the oncogenic properties of human GBM are essential to improve understanding of these tumors and also for testing therapeutics. Work with traditional adherent glioma cell lines is limited because these lines often relate poorly to the histopathology and biologic behavior of tumors in patients [8, 9]. In a broader sense, prolonged passaging, immortalization, and culture conditions have been shown to alter non-glioma adherent models until they are no longer ideally suited for preclinical studies [26, 27]. There may also be changes with continued passage of oncosphere lines and short term oncosphere cultures seem to be the best currently available model for GBM research.

There are numerous reports describing the establishment of oncosphere lines from GBM tumor samples [8–13, 15–17]. With the protocol described here, we were able to establish oncosphere lines from approximately a quarter of the tumors attempted. Given the variability of the techniques of establishment as well as the different assays for stem-like cells, direct comparison among these methods is difficult [28].

It is interesting to note that we were able to establish oncosphere lines that met all of our criteria in only a quarter of the tumors processed. There are numerous possible explanations and sources of bias underlying this finding. GBMs are heterogeneous molecularly, genetically, pathologically, and biologically. It is possible there is something specific regarding the genetic and molecular properties from these 12 patients that allowed their tumor cells to grow *in vitro*. An additional consideration is the location of the tumor tissue harvested. There are likely differences in the ability to form cell lines from tissue obtained from central necrotic regions, contrast enhancing regions, and in the periventricular region where the stem/precursor cells likely reside. Interestingly, although JHU-1016A and JHU-1016B were established from the same patient, they had dramatically different growth properties, highlighting the importance of intratumoral heterogeneity. JHU-1016A was a fast growing line *in vitro*, and while both lines generated tumors in all animals injected, JHU-1016A xenografts resulted in an average survival half that of JHU-1016B. Additionally, harvest techniques, specimen transport, and processing and tissue culture specifics, which vary greatly, also likely impact the establishment rate. These issues highlight some of the challenges in establishing cell lines from heterogeneous tumor tissue.

We used four criteria to evaluate these cell lines including 1) genetic uniqueness using STR profiling; 2) oncosphere formation/self-renewal past passage four; 3) lineage capacity as demonstrated by differentiation; and 4) *in vivo* orthotopic tumor formation. To date, 8 of the cell lines have met all four criteria.

Our first criterion was verification of genetic uniqueness. Cross-contamination or incorrect identification of a cell line occurs with unfortunate frequency. Cross-contamination can happen both among human cell lines and between species. MacLeod *et al*. showed that in a commercial cell line repository, 18% of the cell lines were cross-contaminated [29]. Misidentification of cell lines causes significant difficulties [30–32]. Use of STR profiling confirms the unique nature of each cell line [23]. The kits used in our study included a mouse marker to ensure cell line uniqueness and the absence of interspecies cross-contamination.

Our second criterion was growth as cancer derived oncospheres. These oncospheres provide an *in vitro* three-dimensional model for tumor research. A growing body of research on areas such as metabolism and drug resistance has been done on the physiologic relevance of the three-dimensional sphere structure [33–36]. Rodriquez-Enriquez *et al*. investigated the glycolytic rates in multicellular tumor spheres and compared them to adherent monolayers [36]. They found that spheroids had significantly higher rates of glycolysis and suggested this may provide a better model for understanding tumor metabolism.

Leek *et al*. explored hypoxia-inducible factor 1α (HIF-1α) and its effects on tumor cells independent from angiogenesis by using a spheroid model [35]. They demonstrated that HIF-1α has an inhibitory apoptotic effect in spheroid cells. The three-dimensionality of their spheroid model allowed them to mimic solid tumor conditions in which tumor cells may be a significant distance from the nearest blood vessel. In adherent models, all cells are in direct contact with medium, unlike with spheroids, in which the outer layers of cells are in direct contact with the medium.

Translational research in oncology has benefited from spheroid cultures as they may be a better model for drug resistance. Kobayashi *et al*. examined drug resistance in a murine breast carcinoma line, culturing spheroids from *in vivo* tumors [34]. While the spheroids demonstrated drug resistance, resistance was lost upon dissociation to a single cell suspension, suggesting that cell clustering was important. In addition, the same cells grown as a monolayer did not demonstrate the same degree of drug resistance. Desoize *et al*. proposed that multicellular resistance contributed to tumor drug resistance and that to study it *in vitro* required spheroid tumor models [33]. The layered construction of spheres and the necrotic/quiescent cores provided a physiologically relevant model system.

The ability to form spheroids in and of itself it not necessarily a characteristic of cancer stem cells and in this study we investigated multipotency and *in vivo* tumor formation to begin to further evaluate the stem-like nature of our oncosphere lines. Multipotency, our third criterion, was evaluated in the majority of cell lines. Similar to other studies, our work demonstrates such cultures can be differentiated to express different lineage markers [8, 9, 18]. This helps demonstrate a stem-like state of the cell lines. The primitive state of cancer stem cells has been shown to contribute to radioresistance and chemoresistance [37, 38]. With respect to radiotherapy, Bao *et al* demonstrated that not only did treating GBM xenografts with ionizing radiation enhance the cancer stem cell proportion, those cancer stem cells showed higher activation of DNA damage checkpoint proteins [37]. These results suggested the GBM cancer stem cell population demonstrated radioresistance through activation of DNA damage repair. Breast cancer stem cells, identified as a CD24^-^/CD44^+^ enriched population growing as spheres, showed increased resistance to irradiation when compared to a non-enriched, monolayer culture [39]. Similar work demonstrated the chemotherapy resistant nature of the CD24^-^/CD44^+^ enriched population of breast cancer cells [40].

Our fourth criterion was *in vivo* tumor formation. *In vivo* tumor models play a critical role in cancer research, particularly in new drug testing [41]. Oncospheres have been shown to give rise to *in vivo* tumors that have closer histological characteristics to GBMs than adherent cell lines. Lee *et al*. showed that tumors formed by oncosphere cell lines more closely resembled GBMs than tumors formed by adherent cell lines [9]. Importantly, our oncosphere lines recapitulate one of the most important histologic features associated with treatment failure, the ability to infiltrate normal brain parenchyma. Two lines, JHU-0937 and JHU-1014, have not formed xenografts in nude mice. These cell lines provide the opportunity to further investigate *in vivo* tumorigenicity, providing a counterpoint to the xenograft-forming cell lines.

Of note, in our study we were able to establish oncosphere cell lines for patients with GBM variants. One patient, JHU-0879, had PNET features in the tumor. These features were also found in the xenograft, including increased cell density and a large number of apoptotic bodies, which was suggestive of *c-Myc* amplification. Patient JHH-505 had a GBM that contained an oligodendroglioma component. This cell line efficiently formed intracranial xenografts with characteristics of GBM-O. Model systems of these GBM variants are rare in the literature [15]. The creation of more cell lines from such GBM variants will be useful for learning about how these tumor types develop and investigating possible therapeutic interventions.

## Conclusion

We report our protocol for establishing suspension cell lines directly from intraoperatively obtained tumor tissue. Using this protocol, we established 13 primary human oncosphere cell lines derived from patients with GBM and GBM variants. A large body of research has demonstrated that suspension cell lines are more applicable models for GBM and a valuable resource in GBM research.

## Supporting Information

S1 Table STR profile for the established cell lines. (DOCX)
