# Supplementary material for: Establishment and Biological Characterization of a Panel of Glioblastoma Multiforme (GBM) and GBM Variant Oncosphere Cell Lines
Source: PLoS One. 2016 Mar 30;11(3):e0150271. doi: 10.1371/journal.pone.0150271 (PMC4814135; doi:10.1371/journal.pone.0150271)
Supplement: S1 Table — (DOCX) [file pone.0150271.s001.docx]

|  | AMEL | CSF1PO | D13S317 | D16S539 | D21S11 | D5S818 | D7S820 | TH01 | TPOX | vWA |
| --- | --- | --- | --- | --- | --- | --- | --- | --- | --- | --- |
| JHU-0879 | X, X | 12, 12 | 11, 11 | 11, 11 | 31.2, 33.2 | 11, 12 | 10, 10 | 9, 9 | 8, 8 | 15, 16 |
| JHH-66 | X, X | 10, 12 | 9, 11 | 11, 11 | 30, 32.2 | 12, 12 | 12, 13 | 6, 8 | 8, 8 | 16, 17 |
| JHH-75 | X, X | 11, 12 | 11, 11 | 11, 12 | 31.2, 31.2 | 10, 12 | 8, 10 | 6, 9.3 | 10, 12 | 17, 18 |
| JHH-136 | X, Y | 11, 11 | 12, 12 | 12, 12 | 29, 30 | 10, 13 | 9, 11 | 6, 7 | 8, 9 | 14, 18 |
| JHU-0937 | X, Y | 11, 12 | 11, 13 | 11, 12 | 28, 30 | 11, 12 | 9, 11 | 8, 9 | 8, 8 | 17, 18 |
| JHH-211 | X, Y | 10, 10 | 11, 13 | 12, 12 | 30, 31 | 11, 11 | 11, 12 | 7, 9 | 8, 9 | 16, 17 |
| JHH-227 | X, Y | 10, 12 | 10, 14 | 12, 12 | 30, 30 | 11, 12 | 11, 11 | 6, 9 | 9, 9 | 17, 20 |
| JHH-245 | X, Y | 12, 13 | 10, 10 | 9, 11 | 30, 30 | 10, 14 | 8, 8 | 6, 6 | 8, 10 | 16, 17 |
| JHU-1014 | X, Y | 11, 12 | 12, 12 | 13, 13 | 30, 33.2 | 11, 12 | 10, 10 | 9.3, 9.3 | 8, 8 | 16, 18 |
| JHU-1016A | X, Y | 11, 11 | 12, 14 | 12, 12 | 30, 31.2 | 11, 11 | 9, 12 | 6, 9.3 | 8, 11 | 15, 17 |
| JHU-1016B | X, Y | 11, 11 | 12, 14 | 12, 12 | 30, 31.2 | 11, 11 | 9, 12 | 6, 9.3 | 8, 11 | 15, 17 |
| JHH-505 | X, Y | 11, 11 | 11, 12 | 13, 13 | 28, 31 | 12, 12 | 11, 12 | 9.3, 9.3 | 8, 12 | 15, 19 |
| JHH-520 | X, X | 11, 11 | 11, 12 | 9, 13 | 29, 29 | 11, 11 | 10, 11 | 9, 10 | 8, 8 | 19, 19 |
| HeLa | X, X | 9, 10 | 12, 13.3 | 9, 10 | 27, 28 | 11, 12 | 8, 12 | 7, 7 | 8, 12 | 16, 18 |
| K562 | X, X | 9, 10 | 8, 8 | 11, 12 | 29, 30, 31 | 11, 12 | 9, 11 | 9.3, 9.3 | 8, 9 | 16, 16 |

**Supplemental Table 1. STR profile for the established cell lines**
